# A patient-specific CTA-CFD framework deciphers hemodynamic heterogeneity after fenestrated TEVAR: a pilot study

**DOI:** 10.3389/fbioe.2026.1797348

**Published:** 2026-06-08

**Authors:** Baoyou Zhang, Fan Gao, Jian Wang, Wenpu Zhang

**Affiliations:** 1 Department of Cardiac and Thoracic Vascular Surgery, The First People’s Hospital of Jiashan County, Jiaxing, Zhejiang, China; 2 Department of Simulation Science and Technology, Shaanxi Xinmai Medical Technology Co., Ltd., Xi’an, China; 3 Department of Vascular Surgery, The Second Affiliated Hospital of Zhejiang University School of Medicine, Hangzhou, China; 4 School of Aeronautics and Astronautics, Zhejiang University, Hangzhou, China

**Keywords:** computational fluid dynamics, fenestration, hemodynamics, morphologic analysis, TEVAR

## Abstract

**Background and Objectives:**

Despite the widespread use of fenestrated thoracic endovascular aortic repair (TEVAR), clinical outcomes exhibit considerable heterogeneity whose underlying hemodynamic mechanisms remain poorly understood. This study aimed to establish a patient-specific computational framework integrating postoperative computed tomography angiography (CTA) and computational fluid dynamics (CFD) to quantitatively evaluate morpho-hemodynamic alterations after fenestrated TEVAR.

**Methods:**

Six aortic dissection patients undergoing TEVAR with left subclavian artery (LSA) fenestration (three *in situ*, three *in vitro*) were included. Patient-specific 3D aortic geometries were reconstructed from postoperative CTA. High-fidelity CFD simulations were performed to analyze flow distribution, velocity, time-averaged wall shear stress (TAWSS), and oscillatory shear index (OSI).

**Results:**

Computational analysis revealed that the brachiocephalic trunk was the least affected vessel following TEVAR (median AR_BT_ 0.80, IQR 0.74–0.89). Notable abnormalities included one case of severe LSA stenosis (AR_LSA_ = 5.25, V_LSA, systole_ = 1.15 m/s), and one instance of mild stent-induced proximal LCCA compromise (AR_LCCA_ = 1.27, V_LCCA, systole_ = 1.06 m/s), both demonstrating TAWSS elevation at affected segments. Additionally, one patient exhibited inadequate endovascular recovery in the descending aorta, which received only 42.83% of the total cardiac output. The remaining patients showed no significant hemodynamic abnormalities.

**Conclusion:**

This pilot study establishes a patient-specific computational framework that integrates CTA with CFD to decipher post-intervention morpho-hemodynamic alterations. By quantitatively linking stent-induced geometric changes to adverse hemodynamic phenotypes, the demonstrated methodology explores a mechanistic approach for understanding post-surgical outcome disparities, thereby establishing a computational tool for postoperative evaluation. The clinical utility and predictive value of this tool, however, await validation in larger, prospective cohorts.

## 1 Introduction

Thoracic endovascular aortic repair (TEVAR) has revolutionized the management of thoracic aortic pathologies since its introduction in 1994, establishing itself as a cornerstone treatment with demonstrated safety and efficacy over 3 decades of clinical application (Dake et al., 1994; Liu et al., 2020; Harky et al., 2020). However, the optimal management of the left subclavian artery (LSA) during these procedures continues to generate considerable debate in the vascular surgery community. Initial clinical experience suggested that LSA coverage during TEVAR represented a relatively low-risk intervention, with only sporadic reports of complications such as stroke, spinal cord ischemia (SCI), and left upper extremity ischemia (Kotelis et al., 2009; Si et al., 2014). This perspective was further supported by studies indicating that LSA revascularization might potentially increase cardiopulmonary complications and perioperative stroke rates while offering limited symptomatic improvement (Delafontaine et al., 2019; Ghanem et al., 2021). In contrast, accumulating evidence has demonstrated significant benefits associated with LSA revascularization, including a substantial reduction in 30-day SCI incidence based on a study of 171 patients (Xie et al., 2021), alongside mid-to long-term survival advantages (Nejim et al., 2022; Mandigers et al., 2022; Spath et al., 2024).

The persistent controversy surrounding LSA management is reflected in inconsistent clinical outcomes and conflicting statistical analyses across studies. Our own clinical experience with TEVAR and fenestration techniques has similarly revealed considerable interpatient variability in outcomes (Table 1), likely attributable to heterogeneity in revascularization techniques, individual patient physiology, and surgical execution variations. The absence of precise, patient-specific evaluation methodologies following TEVAR with revascularization appears to be a fundamental limitation contributing to these divergent conclusions in the literature.

**TABLE 1:** Summary of patient information for six cases treated with TEVAR.

Case no.	Gender	Age	BP (mmHg)	Fenestration type	Postoperative adverse symptoms
a	F	71	125/76	In vitro	None
b	M	49	188/108	In vitro	Pulmonary Infection Weak Left Radial Artery Pulse
c	M	62	153/90	In vitro	None
d	M	48	211/121	In situ	Pulmonary Infection
e	F	47	118/68	In situ	None
f	M	55	115/75	In situ	None

Computational fluid dynamics (CFD) has become an indispensable tool in cardiovascular research, enabling detailed hemodynamic analysis in complex disease states (Morris et al., 2016; Sobocinski et al., 2016). Previous applications of CFD in TEVAR research have provided valuable insights into phenomena such as incomplete false lumen thrombosis, identifying key hemodynamic parameters including flow velocity, time-averaged wall shear stress (TAWSS), and oscillatory shear index (OSI) as critical factors influencing distal false lumen remodeling (Schwarz et al., 2023; Kamada et al., 2022). However, a critical gap remains in characterizing the specific hemodynamic impacts of stent-graft implantation on the aortic arch and its major branches. Postoperative adverse symptoms commonly associated with TEVAR, such as perioperative stroke, SCI, and LUEI, can be directly linked to blockages or compromised flow in the left common carotid artery(LCCA) and LSA (Xie et al., 2021; Wang et al., 2024; Li et al., 2021). To address this, a computational framework was developed that integrates postoperative computed tomography angiography (CTA) with patient-specific anatomical reconstruction and computational fluid dynamics (CFD) analysis. This framework mechanistically deciphers stent-induced hemodynamic alterations by directly linking geometric changes to flow phenotypes. By providing quantitative, patient-specific insights, this computational framework demonstrates the potential to establish a new paradigm for postoperative assessment. Its core methodology could, upon further validation, be adapted to a broad range of endovascular procedures and vascular pathologies.

## 2 Methods

### 2.1 Clinical information

This retrospective study included a consecutive cohort of six patients diagnosed with Stanford type B aortic dissection who underwent TEVAR with LSA revascularization (fenestration) at our institution. Patients were included based on the availability of high-quality postoperative CTA scans suitable for detailed three-dimensional reconstruction and computational analysis. The cohort comprised three patients treated with *in situ* fenestration and three with *in vitro* fenestration techniques. All procedures were performed at the First People’s Hospital of Jiashan County between 13 January 2022, and 15 January 2025. The detailed clinical information is illustrated in Table 1. Postoperative CTA images were acquired by the Aquilion ONE 320^−ΔΔCT^ and the voxel size is 0.783 mm × 0.783 mm × 0.625 mm. TEVAR procedures are properly performed for all six patients and no postoperative leakage or stent migration was observed. This study was conducted in accordance with the principles of the Helsinki Declaration and received ethical approval from the institutional ethics committees.

### 2.2 Morphologic extraction and analysis

#### 2.2.1 Morphologic extraction

**FIGURE 1:**
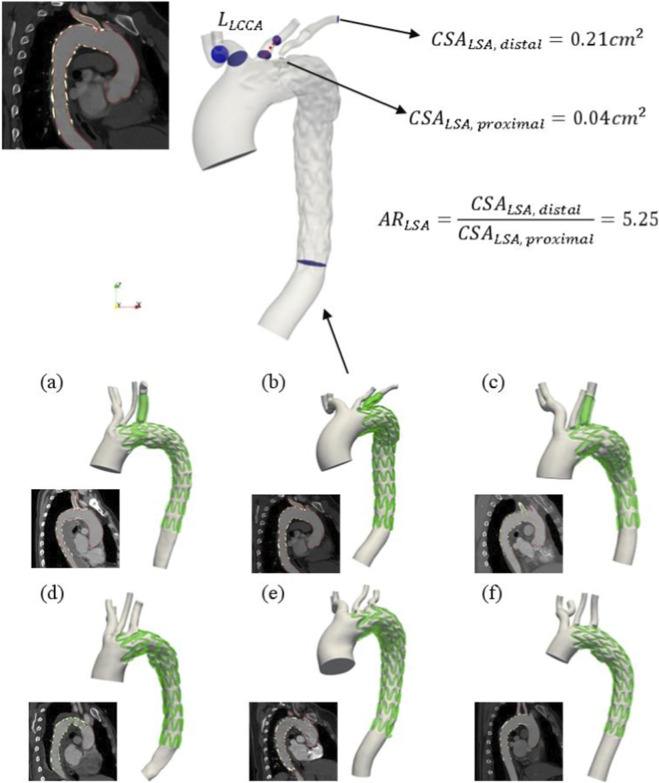
The patient-specific CTA images and 3D geometries of six post-TEVAR patients (Patient **(a–f)**). Patient b is highlighted for the demonstration of applied morphologic analysis in the present study.

**FIGURE 2:**
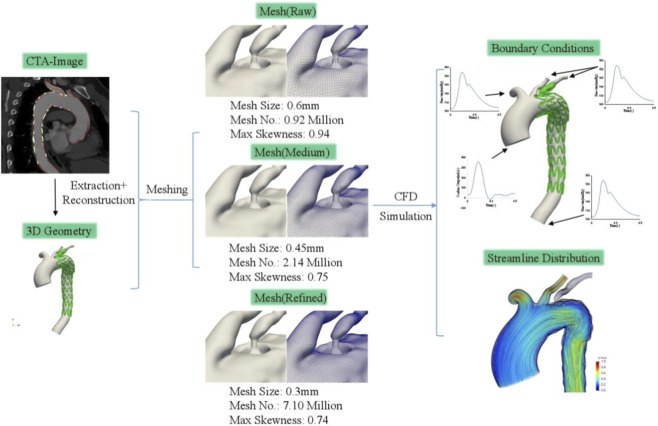
The brief illustration for the mesh generation and CFD analysis process.

As illustrated in Figure 2, the post-TEVAR geometries for six specific patients were reconstructed, with three patients undergoing *in vitro* and *in vivo* procedures respectively. The 3D models of the implanted stents were also extracted and displayed in translucent green. All geometries were created using image voxel luminance extraction (by Mimics) and manually verified to ensure the reconstructed models accurately reflected the patient-specific morphological information provided by the collected CTA images. The scope of the processed 3D models extended from the ascending aorta, as shown in Figure 1. The three major branches of the aortic arch—the brachiocephalic trunk (BT), LCCA, and LSA—were also included in the analysis domain. To ensure the region influenced by TEVAR was fully captured, the distal end of the descending aorta (DA) was also incorporated into the models.

### 2.3 Morphologic analysis

Given that the thoracic aortas in this study were patient-specifically remodeled without evidence of leakage or stent migration, the primary focus was on the impact of the TEVAR procedure on the three major downstream vessels: LCCA, LSA, and DA. To quantitatively assess these effects, the equation illustrated in Figure 1 will be applied which is enlightened by the effective orifice area in aortic stenosis and percent area stenosis in carotid artery assessment:

$${AR}_{LSA}=\frac{{CSA}_{LSA,distal}}{{CSA}_{LSA,proximal}}$$

In which AR represents area ratio, ${CSA}_{LSA,proximal}$ and ${CSA}_{LSA,distal}$ represent the cross sectional area of proximal (the initiation of each branch) and distal (the end of each branch) of LSA, as depicted in Figure 1. The CSA was measured perpendicular to the vessel centerline, ensuring consistent cross-sectional assessment across different segments. The same equation was also applied to other major branches of the aortic arch. Since the distal end of LCCA is out of the CTA image range, a consistent vessel length ( $L_{LCCA}=3cm$ , depicted in Figure 1) is selected for the determination of ${CSA}_{LCCA,distal}$ to avoid the influence of metallic artefacts and ensure measurement reproducibility. In a healthy, tapering vessel, the proximal cross-sectional area is typically larger than the distal area, resulting in an AR≤1. However, the stent blockage of endothelial lumen might significantly reduce the ${CSA}_{LSA,proximal}$ , leading to an elevated AR value. This metric thus enables the quantitative evaluation of TEVAR’s influence on downstream branches.

Generally the reconstruction of thoracic aortic endovascular lumen will timely alleviate visceral, renal, and lower extremity malperfusion for aortic dissection (AD) patients (Plotkin et al., 2021; Grewal et al., 2021). Nevertheless the poorly rebuilt endovascular lumen for distal DA after TEVAR performance is observed in two patients (Patient a & d) which is due to the incomplete false lumen (FL) thrombosis. The CSA of end stent segment ( ${CSA}_{DA,stent}$ , shown in Figure 1) and distal DA ( ${CSA}_{DA,distal}$ ) are also measured and their numerical ratio is also calculated ( ${AR}_{DAA}$ ) to monitor the recovery of distal DA endovascular lumen and its contrast with stent segment. Brachiocephalic trunk (BT) is rarely influenced by the implanted stents and will be considered as a properly functioned branch in the present study.

### 2.4 Meshing and CFD analysis

#### 2.4.1 Computational mesh generation

A high-quality mesh is essential for achieving calculation convergence and reliable results in CFD simulations. Given that the voxel size of the collected CTA images is 0.783 mm × 0.783 mm × 0.625 mm, an initial mesh size of 0.6 mm was used for spatial discretization. However, as shown in Figure 2, the raw mesh failed to adequately capture the morphological details of the reconstructed geometry and did not meet the requirements for mesh independence. To address this, a mesh refinement study was conducted on a representative patient (Patient b) to determine an appropriate mesh resolution for the cohort. The medium and refined meshes with size 0.45 mm and 0.30 mm were implemented on Patient b, resulting in 2.14 and 7.10 million elements to meet the demands of patient-specific CFD analysis. To ensure the computational results were mesh-independent, a refinement study was performed. The analysis compared the volume flow rate through each branch (Figure 4g) and the values of TAWSS and OSI in critical regions (e.g., near stent struts and stenotic areas) across three mesh densities. The 0.30 mm mesh was selected for all subsequent simulations, as the differences in both global flow splits and local WSS-based parameters between this mesh and a finer one were within acceptable tolerances (≤2% for flow split and ≤5% for the peak values of TAWSS/OSI within the stenotic region of LSA), indicating that the solution is mesh independent. Prism boundary layers were implemented on all vessel walls to accurately resolve the near-wall flow. The mesh was configured with three layers and a growth ratio of 1.2 to ensure a dimensionless wall distance (y+) of less than 1, which is essential for the direct resolution of the viscous sublayer and the accurate computation of wall shear stress. All computational meshes were generated using the snappyHexMesh utility within OpenFOAM. Courant number is set as 1.0 for the calculation stablilization and the time-step size is adaptive to this setting (initial time-step size is 0.0005 s). The transient simulations were conducted for three complete cardiac cycles to achieve periodic flow convergence. The normalized residual tolerances for the iterative solution were set to 1 × 10^−6^ for pressure and 1 × 10^−4^ for velocity, respectively. The results from the first two cycles were discarded to eliminate start-up transients, and the flow field from the final cycle was used for all subsequent analyses. The computations were performed on a high-performance computing cluster utilizing a CPU with 48 cores to ensure processing efficiency.

#### 2.4.2 CFD analyses

The hemodynamic simulations in this study were conducted using the open-source CFD library OpenFOAM (OpenCFD Ltd). The PISO (Pressure-Implicit with Splitting of Operators) algorithm was employed to numerically solve the incompressible transient blood flow, with the Navier-Stokes equations serving as the governing equations for the CFD simulation. Blood was modeled as an incompressible, non-Newtonian fluid, with a density of 1060 kg/m^3^. The shear-thinning behavior of blood was modeled using the Carreau model (Akbar and Nadeem, 2014), where the dynamic viscosity μ is a function of the shear rate $\dot{\gamma }$ :

$$\mu =\mu_{\infty }+\left(\mu_{0}-\mu_{\infty }\right){\left[1+{\left(\lambda \dot{\gamma }\right)}^{2}\right]}^{\frac{n-1}{2}}$$

where $\mu_{0}=0.056\text{Pa}\cdot s$ is the zero-shear-rate viscosity, $\mu_{\infty }=0.0035\text{Pa}\cdot s$ is the infinite-shear-rate viscosity, λ=3.313 s is the time constant, and n=0.3568 is the power-law index (Gijsen et al., 1999). Given that this retrospective study is based on routinely acquired postoperative CTA scans, in which advanced patient-specific flow imaging or invasive pressure measurements were not part of the standard clinical care pathway, a well-established, physiologically representative pulsatile inflow waveform and uniform pressure profiles at the outlets were adopted from prior literature on aortic hemodynamics (Vasava et al., 2012; Alastruey et al., 2016). This controlled approach ensures that observed differences in flow distribution and wall shear stress can be attributed with greater confidence to the unique stent-induced geometries of each patient, rather than to inter-individual differences in cardiac output or peripheral resistance, which are difficult to obtain precisely in a retrospective setting. The methodology of using uniformed boundary conditions to focus on geometric comparative analysis is a recognized and valid strategy in hemodynamic studies where the primary aim is to understand the relative impact of morphology under standardized conditions (Qiao et al., 2019; Vignon-Clementel et al., 2010). Furthermore, critical hemodynamic parameters such as peak systolic blood velocity, TAWSS, and oscillatory shear index (OSI), a dimensionless hemodynamic parameter that quantifies the temporal directional variation of WSS throughout the cardiac cycle, were extracted to provide deeper insights into post-TEVAR hemodynamics:

$$TAWSS=\frac{1}{T}\int_{0}^{T}\tau_{w}dt$$

$$OSI=\frac{1}{2}\left(1-\frac{\left|\int_{0}^{T}\tau_{w}dt\right|}{\int_{0}^{T}\left|\tau_{w}\right|dt}\right)$$

In which T and t represents for time, $\tau_{w}$ represents for wall shear stress. The verified blood flow distribution for the BT (19.3%), LCCA (5.2%), LSA (6.4%), and DA (69.1%) based on a healthy individual is treated as a reference in the present study (Vasava et al., 2012).

## 3 Results

### 3.1 Patient-specific morpho-hemodynamic characteristics

**FIGURE 3:**
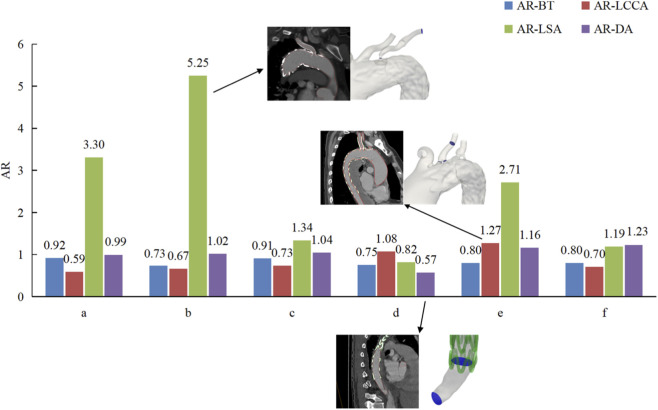
The plot of AR for BT, LCCA, LSA and DA for six post-TEVAR patients. The screenshots of cases with abnormal AR values are also illustrated.

Patient-specific 3D geometries were successfully reconstructed for all six post-TEVAR cases. Quantitative morphologic analysis revealed substantial inter-patient heterogeneity in stent-induced anatomical alterations (Table 2; Figure 3). The brachiocephalic trunk (BT) was the least affected branch (median AR_BT_ 0.80, IQR 0.74–0.89).

**TABLE 2:** The extracted morphologic information for six post-TEVAR patients (Unit: cm^2^).

Case no.	a	b	c	d	e	f
BT_proximal_	1.53	1.46	1.34	1.76	1.17	1.57
BT_distal_	1.40	1.07	1.22	1.32	0.94	1.25
LCCA_proximal_	0.51	0.54	0.56	0.40	0.22⬇	0.54
LCCA_distal_	0.30	0.36	0.41	0.43	0.28	0.38
LSA_proximal_	0.23⬇	0.04⬇	0.50	0.94	0.14⬇	0.52
LSA_distal_	0.76	0.21	0.67	0.77	0.38	0.62
DA_stent_	2.15	3.37	3.36	2.87	2.78	3.51
DA_distal_	2.13	3.44	3.51	1.65⬇	3.23	4.32

**FIGURE 4:**
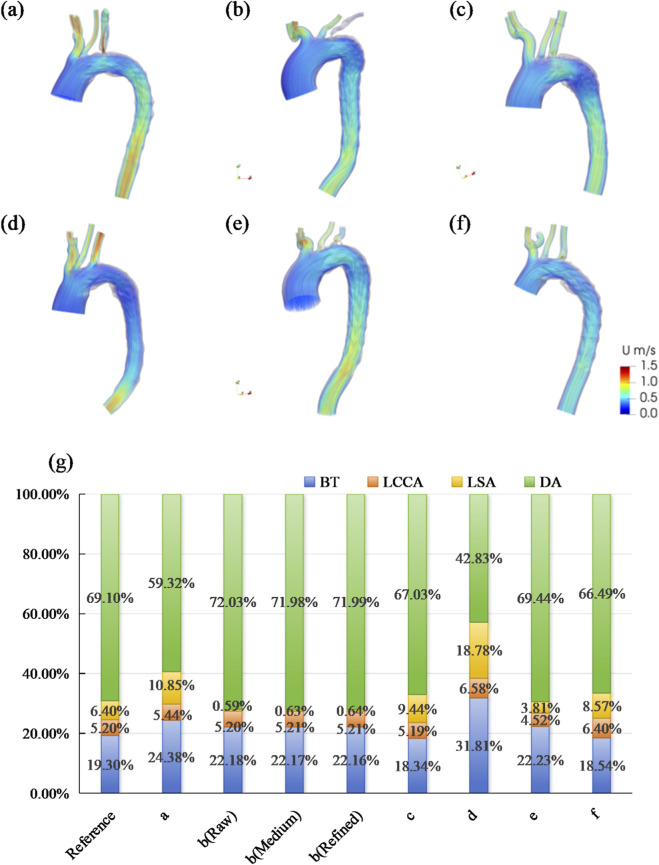
**(a–f)** Streamline distribution for six post-TEVAR patients respectively. **(g)** Proportional volume flow rate distributions for each specific branches during the cardiac cycle. Reference represents for the blood flow stream distribution of healthy individual measured by PCI-MRI.

Notable abnormalities were identified in specific patients, correlating with distinct hemodynamic consequences (Figures 4a–f). Patient b exhibited severe proximal LSA stenosis (AR_LSA_ = 5.25, CSA_LSA, proximal_ = 0.04 cm^2^), which corresponded to a markedly reduced LSA flow (0.64% of total cardiac output) and a clinically weak left radial pulse. Patients d showed inadequate remodeling of the descending aortic true lumen, evidenced by low DA flow ratios and AR_DA_ values of 0.57. Specifically, Patient d displayed profoundly compromised DA perfusion, receiving only 42.83% of the total cardiac output. This integrated morpho-hemodynamic assessment established a direct link between postoperative anatomy and function, identifying patients with quantifiable perfusion deficits for further mechanistic analysis.

### 3.2 Association of geometric alterations with adverse hemodynamic phenotypes

The computed hemodynamic parameters revealed systematic alterations associated with stent-induced geometric changes across the cohort (Table 3). Focal elevations in TAWSS were consistently observed at sites of luminal narrowing, most prominently in the severely stenosed LSA of Patient b (TAWSS_LSA_ = 33.6 Pa). Elevated OSI values, indicating disturbed, oscillatory flow, were identified in specific regions such as the medial aortic arch and downstream of the stenotic LSA segment in Patient b (Table 3(a2)), suggesting the induction of non-physiological flow patterns by the intervention.

**TABLE 3:** (a-f) represent six post-TEVAR patients illustrated in Table 1 respectively, while 1 and 2 represent for TAWSS and OSI distribution respectively.

TAWSS Distribution	*In Situ*	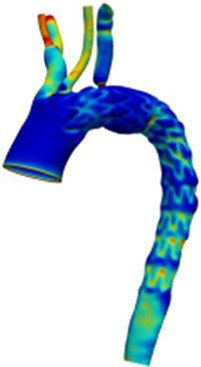	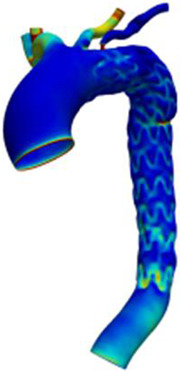	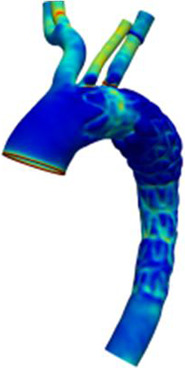
	*In Vitro*	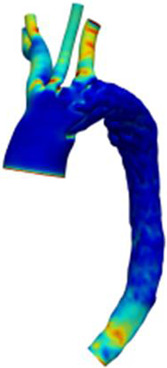	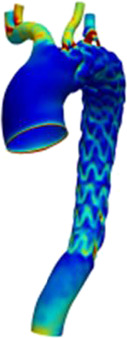	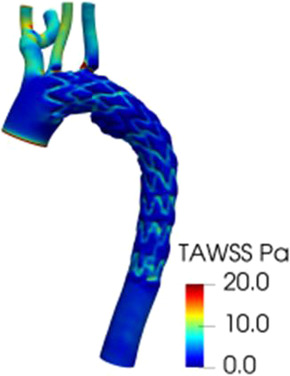
OSI Distribution	*In Situ*	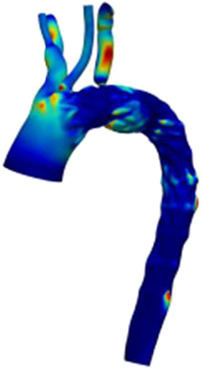	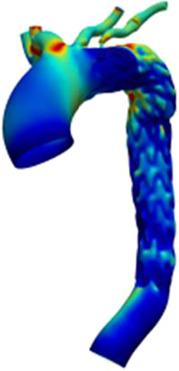	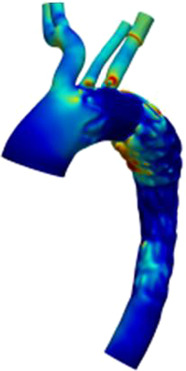
	*In Vitro*	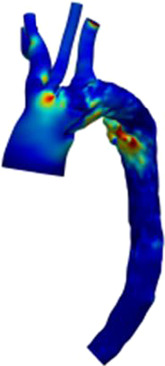	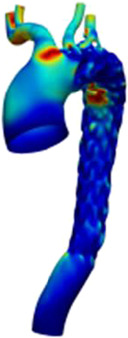	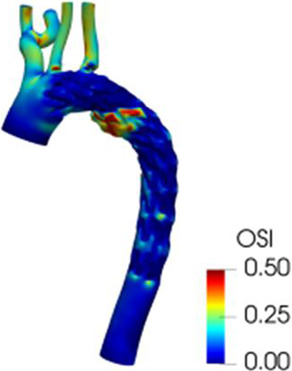

A detailed analysis of patients with marked morphological abnormalities further illustrates the direct structure-function relationship (Table 4). Patient b, who had severe LSA stenosis, exhibited the characteristic high-TAWSS phenotype. The inadequate distal aortic remodeling Patient d demonstrated significantly reduced DA flow (42.83%) alongside elevated distal TAWSS. Patient e (mild LCCA compromise) showed a moderate TAWSS increase at the proximal segment. This spectrum of findings quantitatively links the degree and location of geometric alteration to specific adverse hemodynamic consequences, delineating a high-risk phenotype characterized by pro-thrombotic shear stress and pro-inflammatory flow instability.

**TABLE 4:** Heterogenous morphological and hemodynamic performances after TEVAR with fenestration.

Heterogeneity	Geometry	Velocity	TAWSS
LCCA Interference (Patient e)	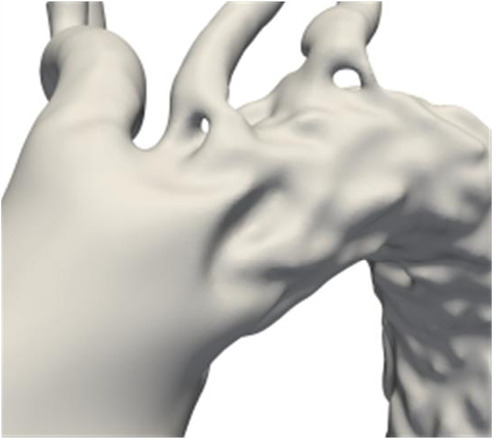	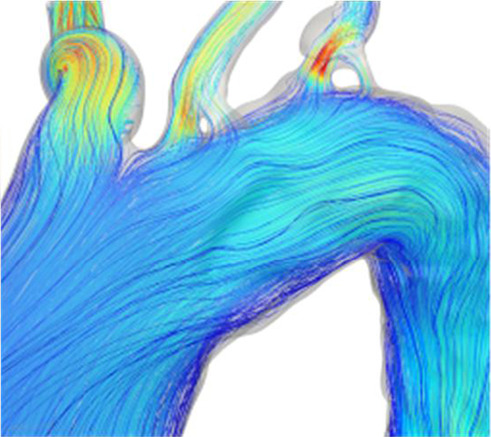	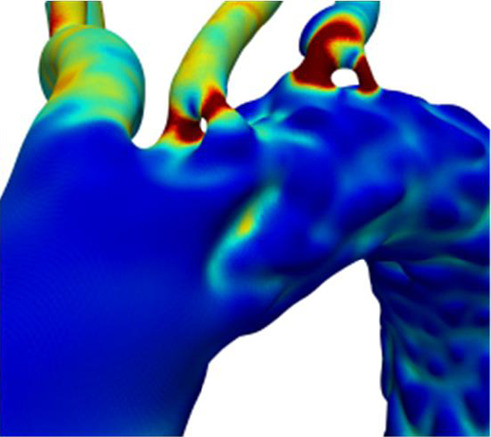
LSA Stenosis (Patient b)	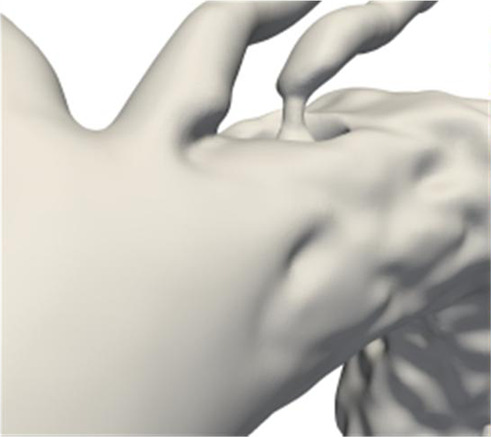	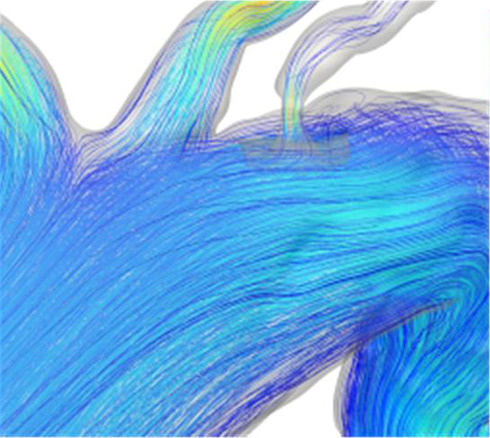	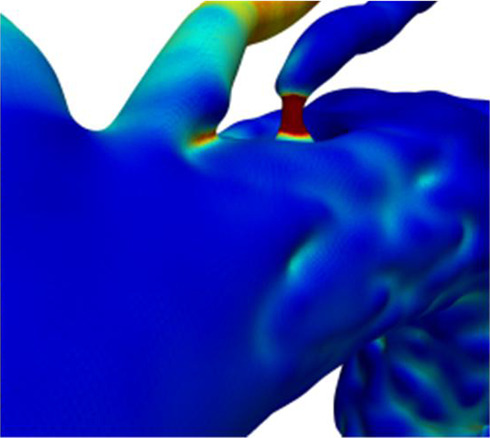
Distal DA Compression (Patient d)	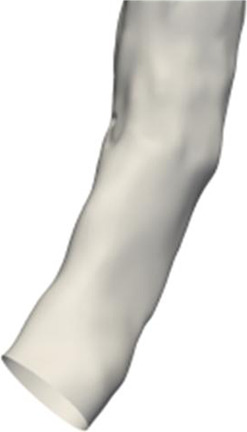	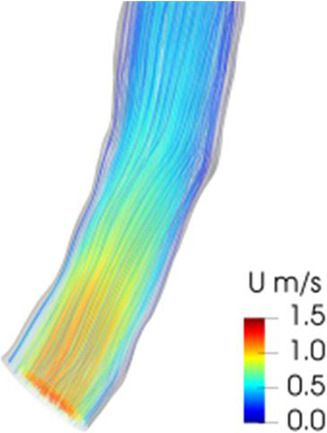	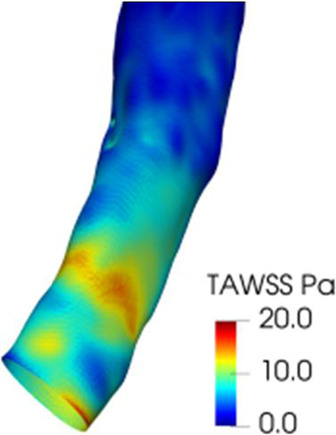

## 4 Discussion

As identified in the introduction, the persistent controversy surrounding optimal LSA management during TEVAR stems from inconsistent clinical outcomes and conflicting statistical analyses across population-level studies (Delafontaine et al., 2019; Ghanem et al., 2021; Rimbau et al., 2017). Such ambiguity likely originates from a fundamental methodological gap: the absence of precise, patient-specific tools to quantify the subtle but decisive morpho-hemodynamic alterations induced by the procedure. The present study tried to address that gap by introducing an integrated computational framework that combines postoperative CTA with CFD analysis. The approach moves beyond traditional anatomical assessment to explore a quantitative, mechanistic basis for understanding the observed heterogeneity in surgical outcomes (Morris et al., 2016; Schwarz et al., 2023).

### 4.1 Quantitative characterization and clinical correlation

Computational analysis revealed that ostensibly successful fenestrated TEVAR procedures produce a spectrum of postoperative morpho-hemodynamic conditions, confirming the clinical observation of interpatient variability in outcomes (Xie et al., 2021). The analytical framework quantified substantial geometric heterogeneity attributable to stent placement, ranging from minimal BT involvement to severe LSA stenosis (Li et al., 2021). Specifically, Patient b exhibited severe proximal LSA stenosis (AR_LSA_ = 5.25) with markedly reduced flow (0.64% of cardiac output), while Patient d showed inadequate distal aortic remodeling with profoundly compromised perfusion (42.83% of cardiac output). The anatomical variations demonstrated direct correlation with distinct hemodynamic consequences (Qiao et al., 2019). Severe stenosis corresponded with marked flow reduction, while compromised distal aortic remodeling resulted in significant perfusion deficits (Wang et al., 2024; Fattori et al., 2013). These findings indicate that even technically adequate procedures can result in quantifiable hemodynamic compromise. The variability in postoperative outcomes could stem from these branches being affected to different extents.

Building upon the quantitative findings, the observed morpho-hemodynamic alterations may be contextualized within the framework of established vascular physiology to suggest clinical correlates. For instance, compromise of the LCCA, as indicated by an elevated AR or adverse local hemodynamics, would primarily affect cerebral perfusion, thereby constituting a theoretical risk factor for perioperative stroke. Similarly, issues affecting the LSA, such as the severe stenosis quantified in Patient b, directly involve the vascular supply to the spinal cord and left upper extremity, which are the recognized anatomical territories for complications like SCI and left arm ischemia. Furthermore, inadequate remodeling or compromised flow in the DA, as observed in Patient d, relates theoretically to the risk of distal malperfusion. This branch-specific interpretive lens offers a potential mechanistic link between the quantified abnormalities and the spectrum of clinical outcomes documented after TEVAR, and may help to contextualize the heterogeneous results reported in the literature. It should be noted that these are explanatory correlations derived from a small pilot cohort, and their predictive value for individual patient outcomes requires prospective validation in larger studies.

These findings contribute to post-interventional hemodynamic understanding by establishing a quantitative methodology to assess the physiological impact of anatomical variations. Such an approach provides a pathway to translate subjective morphological assessments into objective metrics of shear stress and flow stability (Vasava et al., 2012; Alastruey et al., 2016), thereby generating a mechanistic hypothesis for how technically comparable procedures yield divergent physiological outcomes (Tse et al., 2013). The case-specific correlations lend support to the framework’s ability to link anatomy to clinically observable findings. The methodology thus begins to address fundamental questions of clinical utility by suggesting the hemodynamic determinants underlying variable patient responses, offering a different perspective on the inconsistent outcomes reported in clinical studies of endovascular management during TEVAR.

### 4.2 Mechanistic insights and pathophysiological implications

The identified hemodynamic abnormalities represent more than numerical outputs—they correspond to physiological environments with established pathological consequences (Pal, 2021). CFD further identified specific high-risk hemodynamic signatures characterized by localized elevations in TAWSS and OSI at sites of geometric alteration, consistent with previous computational studies of stent-induced flow disturbances (Nauta et al., 2017). The elevated TAWSS in stenotic LSA (33.6 Pa) of Patient b and the disturbed flow patterns indicated by elevated OSI represent quantifiable hemodynamic risk markers that conventional imaging would not capture. The critically elevated TAWSS observed at stenotic sites is recognized in the literature as a pro-thrombotic stimulus (Nobili et al., 2024). In Patient b, the TAWSS of 33.6 Pa at the LSA stenosis far exceeds physiological ranges, creating conditions conducive to platelet activation and potential acute thrombosis. Excessive shear forces are known to activate platelets and induce pro-coagulant endothelial states (van Bakel et al., 2018; Chiu and Chien, 2011). This mechanistic understanding provides a potential hemodynamic correlate for the acute thrombosis risk documented within revascularized branches, which aligns with clinical reports of early post-TEVAR thrombotic complications (Xie et al., 2021).

Equally significant for long-term outcomes, the elevated OSI observed downstream of stent-induced distortions indicates a pro-inflammatory and hyperplastic flow environment (Chiu and Chien, 2011). The non-uniform OSI distribution in Patient b, particularly at the medial aortic arch and downstream of the stenotic LSA segment, suggests the development of oscillatory flow patterns that promote endothelial dysfunction. Oscillatory shear stress disrupts normal endothelial function by promoting adhesion molecule expression and stimulating smooth muscle cell proliferation (Chiu and Chien, 2011). Such conditions are favorable for neo-intimal hyperplasia, potentially leading to restenosis and compromising long-term revascularization patency (Mandigers et al., 2022; Spath et al., 2024). This provides a hemodynamic explanation for why some patients experience late-term revascularization failure despite initially patent repairs.

The primary clinical implication of the framework therefore lies in its ability to identify vulnerable vascular segments—regions characterized not merely by reduced lumen diameter, but by active adverse hemodynamic profiles predisposing to both acute thrombotic events and chronic stenotic complications. By quantifying and highlighting these high-risk phenotypes (e.g., elevated TAWSS combined with high OSI), the framework could provide clinicians with objective data to support the stratification of patients requiring more intensive surveillance or adjunctive medical therapy. This capability may therefore support a future paradigm shift from passive anatomical surveillance to active hemodynamic risk stratification (Sobocinski et al., 2016), potentially informing personalized postoperative management strategies.

### 4.3 Toward personalized management and future directions

The present pilot study introduced a foundational computational methodology with clear translational potential. The use of standardized boundary conditions (as opposed to personalized Windkessel models) was a deliberate choice to isolate the geometric effects of stent placement, which is the central focus of this pilot work. While this methodological approach serves the immediate study goal, the broader clinical validation of the framework remains a necessary future step. The immediate imperative involves prospective validation of the identified hemodynamic risk markers within larger multi-center cohorts to establish robust correlations with clinical endpoints such as stroke and re-intervention rates. Concurrently, the framework’s ability to link anatomy with hemodynamic consequences makes its extension to pre-procedural surgical planning a logical progression. Applying such analysis to preoperative imaging would enable *in silico* comparison of different endovascular strategies, allowing surgeons to select options that optimize postoperative hemodynamics.

The methodology demonstrates inherent generalizability and could be adapted to evaluate more complex aortic arch or thoracoabdominal endovascular reconstructions involving multiple branch vessels. Ultimately, clinical integration requires workflow optimization through enhanced automation and interoperability, potentially evolving into either an open-source research platform or a certified module within clinical imaging workstations. One of the pivotal steps in translating this framework involves acquiring concurrent, patient-specific hemodynamic data (e.g., via Doppler ultrasound and 4D-flow MRI) for rigorous validation of the simulated parameters. Such evolution from postoperative analytical tool to pre- and peri-operative decision-support system represents the critical next phase in translating computational hemodynamic insights into tangible improvements in patient-specific outcomes.

## 5 Limitations

As a pilot feasibility study, this work is subject to several inherent limitations. First, the modest cohort size (n = 6), though suitable for an initial methodological demonstration, limits the statistical generalizability of the findings and underscores the need for validation in larger, prospective cohorts. Second, to decisively isolate the influence of stent-induced anatomical variation—the central focus of this investigation—standardized hemodynamic boundary conditions were applied, as patient-specific flow data were not available in this retrospective setting. While this controlled approach strengthens the causal inference between geometry and hemodynamics, it is acknowledged that the observed hemodynamic variability reflects the interaction between the imposed common conditions and the individual anatomies. The uniform boundary conditions, which do not account for​ patient-specific downstream vascular resistance and compliance, may influence the absolute distribution of flow at the branches. Future studies incorporating individualized boundary conditions would improve physiological realism. Finally, the study establishes correlative links between geometric alterations and adverse hemodynamic phenotypes. The prognostic value of these parameters for clinical endpoints requires prospective evaluation.

## 6 Conclusions

This study establishes a patient-specific computational methodology integrating CTA and CFD that effectively deciphers hemodynamic heterogeneity following fenestrated TEVAR. Computational analysis revealed substantial interpatient variability, from well-remodeled aortic geometries to complications including LSA stenosis and inadequate descending aortic recovery. By quantitatively linking stent-induced anatomical changes to hemodynamic consequences, this approach reveals a strong association between morphological alterations and functional outcomes, providing mechanistic insight into postoperative disparities. The framework presented herein thus provides a methodological basis for gaining mechanistic insight into the outcomes of complex vascular interventions, and its prospective value as a predictive clinical tool warrants validation *in vivo*, larger-scale studies.

## Data Availability

The original contributions presented in the study are included in the article/supplementary material, further inquiries can be directed to the corresponding authors.
